# Associations between chronic widespread pain, pressure pain thresholds, leptin, and metabolic factors in individuals with knee pain

**DOI:** 10.1186/s12891-023-06773-4

**Published:** 2023-08-09

**Authors:** Maria L.E. Andersson, Emelie Thorén, Charlotte Sylwander, Stefan Bergman

**Affiliations:** 1 Spenshult Research and Development Centre, Halmstad, Sweden; 2 Department of Clinical Sciences, Section of Rheumatology, Lund University, Lund, Sweden; 3 School of Business, Engineering and Science, Halmstad University, Halmstad, Sweden; 4 School of Health and Welfare, Halmstad University, Halmstad, Sweden; 5 Primary Health Care Unit, Department of Public Health and Community Medicine, Department of Medicine, Sahlgrenska Academy, University of Gothenburg, Gothenburg, Sweden

**Keywords:** Chronic widespread pain, Pressure pain thresholds, Leptin, Metabolic factors

## Abstract

**Objective:**

The aim was to study associations between chronic widespread pain, widespread pain sensitivity, leptin, and metabolic factors in individuals with knee pain. A secondary aim was to study these associations in a subgroup of individuals with normal BMI.

**Method:**

This cross-sectional study included 265 individuals. The participants were categorised into three different pain groups: Chronic widespread pain (CWP), chronic regional pain (ChRP), or no chronic pain (NCP). The pressure pain thresholds (PPTs) were assessed using computerised pressure algometry. Low PPTs were defined as having PPTs in the lowest third of all tender points. Leptin and metabolic factors such as BMI, visceral fat area (VFA), lipids, and glucose were also assessed.

**Result:**

Sixteen per cent reported CWP, 15% had low PPTs, and 4% fulfilled both criteria. Those who fulfilled the criteria for CWP were more often women, more obese, and had increased leptin levels. In logistic regression, adjusted for age and gender, leptin was associated with fulfilling criteria for CWP, OR 1.015 (95% CI 1.004–1.027, p = 0.008). In logistic regression, adjusted for age and gender, leptin was associated with low PPTs, OR 1.016 (95% CI 1.004–1.029, p = 0.012). Leptin was also associated with fulfilling both criteria, adjusted for age, sex, and visceral fat area (VFA), OR 1.030 (95% CI 1.001–1.060), p = 0.040.

**Conclusion:**

Leptin was associated with fulfilling the combined criteria for chronic widespread pain and low PPTs, even after adjusting for the visceral fat area (VFA). Longitudinal studies are needed to study the causal relationships between leptin and the development of widespread pain.

**Trial registration:**

clinicalTrials.gov Identifier: NCT04928170.

**Supplementary Information:**

The online version contains supplementary material available at 10.1186/s12891-023-06773-4.

## Introduction

Knee pain is an early feature of knee OA, which precedes structural changes within the joint [1]. In individuals with knee pain, it is relatively common to have a more widespread pain, affecting not only the knee joint but the whole body—about one-third of individuals with knee pain report chronic widespread pain [2, 3].

Obesity is reported to be associated not only with OA but also with chronic widespread pain and fibromyalgia [3–5]. Studies have also reported associations between obesity and higher levels of pain intensity and disease activity in patients with low back pain, psoriatic arthritis (PsoA), rheumatoid arthritis (RA), ankylosing spondylitis (AS) and systemic lupus erythematosus (SLE) [6–9].

Adipose tissue synthesises hormones (adipokines), cytokines, and growth factors [10]. The adipokine leptin was identified in 1994 and is synthesised by mature adipocytes [11, 12]. Leptin regulates hunger and appetite but has other functions, such as glucose tolerance, insulin sensitivity, and immune cell functions [13, 14]. Leptin has recently been reported to be able to act via the sympathetic nervous system (SNS), and leptin receptors have been identified on dorsal root ganglia neurons in the SNS [15]. The SNS regulates leptin synthesis and secretion and modulates leptin levels independent of adiposity [16–18]. The SNS also suppresses pain by descending inhibition of nociceptive transmission in the spinal cord [19–21].

Leptin is increased in individuals with OA, and women have higher levels than men [22–24]. It is associated with OA and symptoms such as pain; however, the mechanisms are not yet known in detail [25, 26]. Leptin has further been suggested to be involved in the pathogenesis of OA, up-regulating matrix metalloproteinases (MMPs), and contribute to the low-grade inflammation seen in OA [22, 24].

Pain and allodynia are significant symptoms in patients with lipedema, and they have increased levels of leptin and achieve pain relief after liposuction [27]. Some studies also report improvement in pain intensity and sensitisation after weight loss in the general population as well as in individuals with musculoskeletal diseases [28–31]. Increased metabolic risk factors have been reported also in individuals with normal BMI [32, 33]. However, knowledge of the pathophysiological mechanisms involved in pain sensitisation and widespread pain is still scarce.

The aim was to study associations between chronic widespread pain, widespread pain sensitivity, leptin, and metabolic factors in individuals with knee pain. A secondary aim was to study the associations also in a subgroup of individuals with normal BMI.

## Method

### Participants

In total, 265 individuals with complete assessment for pressure pain thresholds (PPTs) from the Halland osteoarthritis cohort (HALLOA), ClinicalTrials.gov NCT04928170, were included in this cross-sectional study. HALLOA is a longitudinal cohort study including individuals with knee pain at baseline [34]. The inclusion criteria for the HALLOA cohort were current knee pain, with no former known radiographic knee OA and no cruciate ligament rupture or rheumatological disorder, with a preferable age of 30–65 years. The mean age and standard deviation (SD) were 51.6 (8.8), and 71% were women.

### Outcome measures

#### Chronic pain assessment and classifications

Participants marked their painful areas, if they had any, on a pain figure that showed 18 predefined regions [35]. They were categorised into three different pain groups by the modified WP2019 definition [36]: Chronic widespread pain (CWP), chronic regional pain (ChRP) if criteria for CWP were not met, or no chronic pain (NCP). The definition of WP2019, where the knees were excluded, was used due to higher goodness of fit.

The pressure pain thresholds (PPTs) were measured on eight of the 18 predefined tender points by the definition of fibromyalgia [37]. The locations of the eight tender points were: trapezius (bilateral, midpoint of the upper border); second rib (right side, at the second costochondral junctions, just lateral to the junctions on the upper surfaces); lateral epicondyle (right side, 2 cm distal to the epicondyles); knees (bilateral, at the medial fat pad proximal to the joint line); and gluteus (bilateral, in upper outer quadrants of the buttocks in the anterior fold of the gluteus maximus muscle). A hand-held pressure algometer with a 1 cm^2^ rubber probe and a computer interface with an assistant linear response to force application (AlgoMed, Medoc, Ramat Yishai, Israel) were used. A description of the pressure pain threshold assessment has been published previously [3]. Individuals with a PPT value in the lowest third in all separate tender points were included in the group with low PPTs to study those with a more widespread pain sensitivity.

#### Clinical assessments of metabolic factors

The participant’s height and weight were measured, and BMI was calculated. Visceral fat area (VFA) was assessed by bioimpedance measurement, Inbody 770 (Biospace South Korea).

Fasting plasma glucose (mmol/L), triglycerides (TG) (mmol/L), total cholesterol (mmol/L), high-density lipoprotein (HDL)-cholesterol and low-density lipoprotein (LDL)-cholesterol (mmol/L), haemoglobin A1c (HbA1c) (mmol/mol) and C-reactive protein (CRP) > 1.0 mg/L were measured from venous blood, by the current laboratory standards at Halland County Hospital in Halmstad, Sweden, accredited according to SS-EN ISO 15,189. Glucose, TG, total cholesterol, HDL- and LDL-cholesterol were analysed with photometry (Cobas 8000, Roche). HbA1c was analysed with liquid chromatography (TOSOH G8), and CRP was analysed with turbidimetry (Cobas 8000, Roche). CRP below 1.0 mg/L was further explored by applying a sensitive CRP ELISA method (Abnova). Fasting serum leptin was analysed with an enzyme-linked immunosorbent Assay (ELISA) method (Alpco). The leptin analyses were performed as duplicates. Raised glucose was classified as fasting plasma glucose ≥ 5.6 mmol/L or previously diagnosed type II diabetes [38]. Raised triglycerides were classified as triglycerides ≥ 1.7 mmol/L or having specific treatment for this lipid abnormality [38]. Reduced HDL-cholesterol was classified as HDL-cholesterol < 1.03 mmol/L in males and 1.29 mmol/L in females, or specific treatment for this lipid abnormality [38].

### Statistics

To test the differences between groups, the Mann-Whitney U test and the chi-squared tests were used, where appropriate. If comparisons of more than two groups were performed, the Kruskall-Wallis test was used, and if p < 0.05, pairwise comparisons were performed. The missing data were not replaced. All missing data were addressed in the tables. In the analysis of self-reported pain distribution, the three pain groups, NCP, ChRP, and CWP, were compared. In the logistic regression models, CWP was compared to not having CWP as the dependent variable. In the analysis of bilateral PPTs (trapezius, knees, and gluteus), the mean of the two bilateral PPTs on each tender point was used [39]. To study those with more widespread pain sensitivity, those with a PPT value in the lowest third in all separate tender points were included in the group with low PPTs. Low PPTs were compared to not having low PPTs as the dependent variable in the logistic regression models. Variables associated with reporting CWP or having low PPTs, respectively, with a significance level of p ≤ 0.25, were introduced into a multivariate model [40], controlling for age and sex. Separate logistic regression models were also performed between leptin, CWP and low PPT,s adjusting for age, sex and VFA. Dichotomised variables of glucose, triglycerides, and HDL-cholesterol, according to the International Diabetes Federation, were chosen, taking medical treatment into account [38].

Analyses of subgroups were performed by selecting individuals with a normal BMI (BMI < 25) and comparing the groups NCP, ChRP, and CWP and the groups with or without Low PPTs, respectively. Analyses of the combined groups, which fulfilled either or both criteria (CWP and/or low PPTs), were also performed. The logistic regression analysis was performed using the group fulfilling both criteria (Low PPTs and CWP) compared to not doing so as the dependent variable. This group was seen as the most fibromyalgia-like group.

The significance tests were two-tailed and conducted at the 0.05 level of significance. Statistical analyses were performed using SPSS version 21.0 statistical software (IBM Corp., Armonk, NY, USA).

## Results

### Chronic widespread pain and associations with metabolic factors

Out of 265 included individuals, 250 individuals answered the questionnaire, the mean (sd) age was 51.6 (8.8) years, and 71% were women. There were no significant differences between those who answered the questionnaire and those who did not in age, sex, BMI, VFA, and leptin. Of those who answered the questionnaire, 16% reported CWP. They were more often women and had increased BMI compared to NCP (p = 0.014) and higher VFA compared to both NCP (p = 0.001) and ChRP (p = 0.009). Those reporting CWP had more often raised triglycerides (standard residual 2.5) and higher leptin levels compared to both NCP (p = 0.002) and ChRP (p = 0.001), Table 1.

**Table 1:** Description and comparisons of those reporting NCP, ChRP, and CWP, respectively

	Missing	NCP Median (IQR)	ChRP Median (IQR)	CWP Median (IQR)	p-value
N		48	162	40	
Age, year	0/0/0	56.5 (14)	54.0 (10)	53.0 (11)	0.757
Sex, female n (%)	0/0/0	30 (62)	111 (68)	36 (90)	0.010
BMI, kg/m^2^	1/1/1	23.0 (3.8)	25.6 (5.6)	27.4 (7.7)	0.025
VFA, cm^2^	1/3/1	65 (31)	98 (64)	134 (85)	0.001
HbA1c, mmol/mol	2/6/0	36 (5)	37 (5)	38 (4)	0.304
Glucose, mmol/L	0/1/1	5.4 (0.7)	5.4 (0.7)	5.4 (0.8)	0.964
Raised glucose* n (%)	0/1/1	18 (38)	57 (35)	15 (38)	0.921
Triglycerides, mmol/L	0/0/0	0.8 (0.5)	0.9 (0.5)	1.1 (0.9)	0.027
Raised triglycerides* n (%)	0/0/0	5 (10)	20 (12)	12 (30)	0.012
Cholesterol, mmol/L	0/0/0	5.4 (1.6)	5.1 (1.3)	5.2 (1.7)	0.136
HDL-Cholesterol, mmol/L	0/0/0	1.8 (0.8)	1.6 (0.6)	1.6 (0.5)	0.470
Reduced HDL-Cholesterol* n (%)	0/0/0	4 (8)	19 (12)	8 (20)	0.232
LDL-Cholesterol, mmol/L	0/0/0	3.6 (1.3)	3.3 (1.3)	3.2 (1.7)	0.308
Leptin, ng/ml	0/0/0	8.7 (15.6)	13.7 (22.2)	23.8 (59.5)	0.002
CRP, mg/L	0/1/0	1.0 (0.9)	1.1 (1.4)	1.4 (1.7)	0.176

Body mass index, BMI; visceral fat area, VFA; haemoglobin A1c, HbA1c; high-density lipoprotein, HDL; low-density lipoprotein, LDL; C-reactive protein, CRP; knee injury and osteoarthritis outcome, KOOS

*According to International Diabetes Federation [38]

Both univariate and multivariate regression models, adjusted for age and sex, showed associations between female sex, obesity (BMI and VFA), raised triglycerides, increased leptin, and CWP, Table 2. The correlation between VFA and leptin was r = 0.74, p < 0.001. When adjusting for age, sex, and VFA, leptin and CWP showed no significant association with OR 1.007 (95% CI 0.989–1.025, p = 0.461).

**Table 2:** Associations with CWP analysed with two logistic regression models. The multivariate logistic regression model was adjusted for age and sex

	Univariate			Multivariate			
	OR	95% CI	p-value	OR	95% CI	p-value	
Age, year	1.016	0.974–1.058	0.464				
Sex, female n (%)	4.404	1.507–12.871	0.007				
BMI, kg/m^2^	1.082	1.013–1.156	0.020	1.085	1.016–1.160	0.015	
VFA, cm^2^	1.010	1.004–1.016	0.001	1.009	1.002–1.015	0.007	
Raised triglycerides*	3.171	1.432–7.022	0.004	3.864	1.656–9.018	0.002	
Leptin, ng/ml	1.020	1.009–1.031	< 0.001	1.015	1.004–1.027	0.008	

Body mass index, BMI; visceral fat area, VFA; haemoglobin A1c, HbA1c; high-density lipoprotein, HDL; low-density lipoprotein, LDL; C-reactive protein, CRP

*According to International Diabetes Federation [38]

In a subgroup with BMI < 25 (n = 115), 12% reported CWP, Table 3. Also, in this subgroup, both VFA and leptin, adjusted for age and sex, were associated with CWP (VFA OR 1.021, 95% CI 1.001–1.040, p = 0.035 and leptin OR 1.036, 95% CI 1.002–1.072, p = 0.038), supplement Table 1. The correlation between VFA and leptin in this subgroup was r = 0.68, p < 0.001. Leptin and CWP were not significantly associated when adjusted for age, sex, and VFA, OR 1.019(95% CI 0.970–1.070, p = 0.449).

**Table 3:** Subgroup analysis of individuals with normal BMI (BMI < 25 kg/m2). Description and comparison of those reporting NCP, ChRP, and CWP

	Missing	NCP Median (IQR)	ChRP Median (IQR)	CWP Median (IQR)	p-value
N		27	74	14	
Age, year	0/0/0	53 (15)	53 (13)	52 (15)	0.992
Sex, female n (%)	0/0/0	19 (70)	59 (80)	13 (93)	0.238
BMI, kg/m^2^	0/0/0	22.9 (2.4)	23.0 (2.8)	23.0 (1.7)	0.724
VFA, cm^2^	1/1/0	63 (21)	73 (28)	80 (37)	0.049
HbA1C, mmol/mol	1/2/0	36 (4)	36 (4)	35 (5)	0.632
Glucose mmol/L	0/1/0	5.1 (0.6)	5.2 (0.6)	5.2 (0.5)	0.906
Triglycerides, mmol/L	0/0/0	0.8 (0.3)	0.8 (0.4)	0.7 (0.7)	0.359
Raised triglycerides*, n (%)		0 (0)	1 (1)	3 (21)	< 0.001
Cholesterol, mmol/L	0/0/0	5.1 (2.0)	5.0 (1.0)	4.6 (1.4)	0.251
HDL-Cholesterol, mmol/L	0/0/0	2.0 (0.9)	1.8 (0.5)	1.6 (0.9)	0.166
Reduced HDL-Cholestero*l, n (%)		1 (4)	3 (4)	3 (21)	0.037
LDL-Cholesterol, mmol/L	0/0/0	3.3 (1.5)	3.0 (1.5)	3.0 (1.2)	0.333
Leptin, ng/mL	0/0/0	6.0 (7.6)	9.6 (11.)	8.5 (18:8)	0.203
CRP, mg/L	0/0/0	0.7 (0.8)	0.7 (0.7)	1.1 (1.7)	0.467

Body mass index, BMI; visceral fat area, VFA; haemoglobin A1c, HbA1c; high-density lipoprotein, HDL; low-density lipoprotein, LDL; C-reactive protein, CRP

*According to International diabetes federation (IDF) [38]

### Pain pressure thresholds and associations with metabolic factors

Fifteen per cent of the included individuals had low PPTs. The individuals with low PPTs were younger, had higher VFA, more often reduced HDL-cholesterol, and increased leptin Table 4.

**Table 4:** Description of those who did not have low PPT compared to those with low PPT

	Missing Not low/low	Not Low PPT Median (IQR)	Low PPT Median (IQR)	p-value
N		226	39	
Age, year	0/0	54 (10)	51 (12)	0.045
Sex, female n (%)	0/0	162 (72)	26 (67)	0.524
BMI, kg/m^2^	0/0	25.2 (5.9)	27.5 (5.6)	0.073
VFA, cm^2^	5/0	95 (70)	130 (72)	0.027
HbA1C, mmol/mol	7/2	36 (5)	36 (5)	0.863
Glucose, mmol/L	2/0	5.4 (0.7)	5.4 (0.6)	0.975
Raised glucose* n (%)	2/0	78 (35)	15 (38)	0.661
Triglycerides, mmol/L	0/0	0.9 (0.5)	0.9 (0.7)	0.641
Raised triglycerides* n (%)	0/0	30 (13)	9 (23)	0.111
Cholesterol, mmol/L	0/0	5.2 (1.4)	4.9 (1.4)	0.059
HDL-Cholesterol, mmol/L	0/0	1.7 (0.6)	1.5 (1.7)	0.011
Reduced HDL-Cholesterol* n (%)	0/0	22 (10)	9 (23)	0.017
LDL-Cholesterol, mmol/L	0/0	3.5 (1.4)	3.2 (1.3)	0.197
Leptin, ng/ml	0/0	13.3 (22.8)	22.2 (30.8)	0.016
CRP, mg/L	1/0	1.1 (1.2)	1.3 (2.4)	0.575

Body mass index, BMI; visceral fat area, VFA; haemoglobin A1c, HbA1c; high-density lipoprotein, HDL; low-density lipoprotein, LDL; C-reactive protein, CRP; knee injury and osteoarthritis outcome, KOOS

*According to International Diabetes Federation [38]

Univariate and multivariate logistic regressions, adjusted for age and sex, showed associations between low PPTs and younger age, increased VFA, reduced HDL-cholesterol, and increased leptin, Table 5. The correlation between VFA and leptin was r = 0.75, p < 0.001. There was no significant association between leptin and CWP when adjusted for age, sex, and VFA, OR 1.010 (95% CI 0.992–1.029, p = 0.267).

**Table 5:** Associations with low PPT analysed with two logistic regression models. The multivariate logistic regression model was adjusted for age and sex

	Univariate			Multivariate*		
	OR	95% CI	p-value	OR	95% CI	p-value
Age, year	0.961	0.927–0.997	0.032			
Sex, female n (%)	0.790	0.382–1.633	0.525			
BMI, kg/m^2^	1.043	0.976–1.114	0.215	1.057	0.987–1.131	0.111
VFA, cm^2^	1.005	0.999–1.011	0.090	1.007	1.001–1.014	0.022
Raised triglycerides*	1.960	0.848–4.532	0.116	2.125	0.899–5.024	0.086
Reduced HDL-Cholesterol*	2.782	1.171–6.607	0.020	2.627	1.092–6.320	0.031
Leptin, ng/mL	1.010	0.999–1.022	0.065	1.016	1.004–1.029	0.012

Body mass index, BMI; visceral fat area, VFA; haemoglobin A1c, HbA1c; high-density lipoprotein, HDL; low-density lipoprotein, LDL; C-reactive protein, CRP

*According to International Diabetes Federation [38]

In a subgroup with BMI < 25 (n = 119), 11% had low PPTs, Table 6. In this subgroup, leptin, adjusted for age and sex, was associated with low PPTs (OR 1.030 (95% CI 0.997–1.065), p = 0.073), supplement Table 2. The correlation in this subgroup between VFA and leptin was r = 0.68, p < 0.001. When adjusting for age, sex, and VFA, the association between leptin and low PPT was non-significant, OR 1.010 (95% CI 0.960–1.062, p = 0.669).

**Table 6:** Subgroup analysis of individuals with normal BMI (BMI < 25 kg/m^2^). Description of those who did not have low PPT compared to those with low PPT

	Missing Not low/low	Not low PPT Median (IQR)	Low PPT Median (IQR)	p-value
n		106	13	
Age, year	0/0	53 (14)	49 (17)	0.106
Sex, female n (%)	0/0	85 (80)	10 (77)	0.782
BMI, kg/m^2^	0/0	22.8 (2.6)	23.0 (2.1)	0.600
VFA, cm^2^	2/0	70 (26)	74 (50)	0.521
HbA1C, mmol/mol	3/0	36 (4)	36 (7)	0.989
Glucose, mmol/L	1/0	5.2 (0.6)	5.1 (0.5)	0.976
Triglycerides, mmol/L	0/0	0.8 (0.4)	0.7 (0.4)	0.494
Raised triglycerides n (%)	0/0	2 (2)	2 (15)	0.011
Cholesterol, mmol/L	0/0	5.0 (1.2)	4.4 (1.2)	0.090
HDL-Cholesterol, mmol/L	0/0	1.8 (0.6)	1.6 (1.6)	0.372
Reduced HDL-Cholesterol n (%)	0/0	5 (5)	2 (15)	0.123
LDL-Cholesterol, mmol/L	0/0	3.1 (1.2)	2.6 (1.0)	0.106
Leptin, ng/mL	0/0	7.2 (9.2)	13.8 (18.4)	0.250
CRP, mg/L	0/0	0.8 (0.7)	0.6 (1.0)	0.630

Body mass index, BMI; visceral fat area, VFA; haemoglobin A1c, HbA1c; high-density lipoprotein, HDL; low-density lipoprotein, LDL; C-reactive protein, CRP

*According to International Diabetes Federation [38]

### Leptin levels in those who did or did not fulfil the combined pain criteria

The individuals that fulfilled both criteria (for CWP and low PPTs) were younger than those reporting CWP (p = 0.047). Those fulfilling both criteria had higher VFA and leptin levels than those that did not fulfil the criteria (no pain) (p = 0.028 and p = 0.012), supplement Table 3. Younger age, increased VFA, raised TG, reduced HDL-cholesterol, and leptin was associated with fulfilling both criteria, supplement Table 4. Leptin was associated with fulfilling both criteria, adjusted for age, sex, and VFA, OR 1.030 (95% CI 1.001–1.060), p = 0.040.

## Discussion

This study found that more than every fourth individual with knee pain reported CWP and/or low PPTs and that 4% fulfilled both criteria. Obesity, leptin, and metabolic factors were associated with both CWP and low PPTs.

The link between chronic pain and OA is not fully known. There are studies suggesting mechanical load could induce chronic pain due to compression and aberrant loading [41–43]. In the present study, 11–12% of those with normal BMI (BMI < 25 kg/m2) reported CWP and/or had low PPTs, and in those individuals, the mechanical load should not have a significant impact. There are studies suggesting that inflammation due to obesity could cause chronic pain [42, 44]. However, in the current study, there was no association between reported CWP or having low PPTs with inflammation assessed with CRP. Although, it could be a limitation that the low-grade inflammation in the present study was assessed by CRP and not IL-6, which could be a better biomarker for low-grade inflammation [14].

In the present study, leptin level was increased and associated with CWP and having low PPTs. However, in the sub-studies of normal BMI, there were no significant differences in leptin levels between pain groups (NCP, ChRP and CWP; not low PPTs and low PPTs), but still an association between leptin and CWP. Interestingly, there was an increasing level of leptin, with the lowest level in the no pain group and the highest level in the group that met both criteria. There was an association between fulfilling both criteria and leptin, adjusted for age, sex, and VFA. This study does not reveal if increased leptin is a marker for obesity or has a more active role within the pathophysiological mechanism of chronic widespread pain. There is a high correlation between VFA and leptin, and when adjusting for VFA, an actual association may be removed. In a previous longitudinal study of the transition between pain groups in patients with rheumatoid arthritis, a transition from CWP to NCP was associated with a normal BMI and a transition from NCR/ChRP to CWP was associated with central obesity [45]. In mice, it has been shown that when injecting leptin, the pain behaviour increases and this was reversed with the administration of a leptin agonist [46, 47].

In those with normal BMI, leptin was also increased and/or associated with reporting CWP. There are reports of a new concept of normal-weight obesity or metabolically obese normal-weight individuals. It is defined by normal weight and BMI but with a cluster of metabolic characteristics, such as increased insulin sensitivity, triglycerides, and VFA [33, 48]. In another study within the HALLOA cohort, associations between metabolic factors and OA have also been reported in individuals with normal BMI [32]. A group of individuals within the study could have a metabolic condition, including increased leptin levels despite normal BMI. It was thus essential to test the hypothesis also in the subgroup with normal BMI to reveal if leptin could be associated with CWP and low PPTs also in this subgroup. In a clinical setting, a normal BMI may not be a guarantee that there are no metabolic issues.

Other studies have reported increased leptin levels in migraine [49], pelvic pain in endometriosis [50], lipedema [27], low back pain [51], and multisomatoform disorder (MSD) [52]. Leptin levels have been reported to be reduced when weight is lost [53]. Studies also report symptom and pain reduction in relation to losing weight, not only in OA but also in chronic pain conditions, such as fibromyalgia [4]. In rodents, leptin has been shown to decrease pain thresholds and contribute to neuropathic pain [46, 47]. One study showed that low leptin levels were associated with clinical improvement, assessed by Ankylosing Spondylitis Disease Activity Score (ASDAS) in axSpA patients treated with anti-tumor necrosing factor injections (TNFi) [54]. ASDAS includes pain scores; an improvement in pain scores decreases the total score. The study concluded that obese patients should be encouraged to lose weight.

Leptin is shown to reduce norepinephrine (noradrenaline) concentrations in the paraventricular nucleus, arcuate nucleus, and ventromedial hypothalamus in mice [55]. Noradrenaline is the main neurotransmitter released by the SNS and is involved in the intrinsic control of pain [56, 57]. There is a possibility that leptin could influence the descending control of pain, as the descending control of pain involves norepinephrine [16, 56, 57]. Leptin concentration is associated with the amount of white fat mass, and weight reduction is shown to reduce leptin levels and symptoms such as pain [16, 28, 29, 31, 53, 58–60].

There were also increased levels and associations of other metabolic factors. The patients who reported CWP had higher levels of triglycerides, and triglycerides were associated with reporting CWP, also in the subgroup with normal BMI. Triglycerides were also associated with having low PPTs. The current study found associations between having low PPTs and reduced HDL-cholesterol. Other studies have reported associations between hyperlipidemia, chronic pain, and pain sensitisation [61, 62].

Weight loss is not a high-priority recommendation in treatment recommendations for OA and chronic pain/fibromyalgia [63–65]. Given that obesity and/or increased VFA is a risk factor for both OA and chronic pain/fibromyalgia, weight reduction should have a more prominent position as a treatment option in OA to prevent pain sensitisation and treat chronic pain/fibromyalgia.

The limitation of the current study is its cross-sectional design and not longitudinal; studies of casual relationships are needed. However, as this study is part of a longitudinal cohort study within the HALLOA cohort, causal relationships could be studied in the future. CWP was self-reported, which could be a limitation. The pain figure used for reporting CWP has been used in several studies [2, 35, 66, 67]. The lack of a control group could be a limitation. However, the comparisons between pain groups are valid within this context of individuals with knee pain, giving a possible risk marker for widespread pain. The PPTs assessment was performed by more than one test leader, which could be a limitation, but all the test leaders had previous experience and had undergone training. A previous study shows that at least one hour of training improved reliability [68]. The study’s strength is that it is a population-based cohort with knee pain at baseline and is part of a longitudinal cohort study. Many of the included individuals (76%) have no radiographic knee OA and are considered individuals in the early disease course [32].

In conclusion, leptin and metabolic factors were associated with both CWP and low PPTs in this study. The pathophysiological mechanism causing widespread pain and pain sensitivity is probably multifactorial, involving biological and physical factors. The adipokine leptin could be involved in some of these mechanisms, but longitudinal studies are needed to explore causal relationships and pathophysiological mechanisms. There is also a need to study leptin in other diseases with a risk of developing chronic widespread pain.

### Electronic supplementary material

Below is the link to the electronic supplementary material.

Supplementary Material 1

Supplementary Material 2

Supplementary Material 3

Supplementary Material 4

Supplementary Material 5

## Data Availability

The dataset used and analysed during the current study is available from the corresponding author upon reasonable request.

## Abbreviations

ADL: Function in daily living
AS: Ankylosing spondylitis
BMI: Body mass index
ChRP: Chronic regional pain
CRP: C-reactive protein
CWP: Chronic widespread pain
ELISA: Enzyme-linked immunosorbent assay
HALLOA: Halland osteoarthritis cohort
HDL: High-density lipoprotein
LDL: Low-density lipoprotein
NCP: No chronic pain
OA: Osteoarthritis
PsoA: Psoriatic arthritis
PPTs: Pressure pain thresholds
RA: Rheumatoid arthritis
SLE: Systemic lupus erythematosus
SNS: Sympathetic nervous system
VFA: Visceral fat area
