# Supplementary material for: Associations between chronic widespread pain, pressure pain thresholds, leptin, and metabolic factors in individuals with knee pain
Source: BMC Musculoskelet Disord. 2023 Aug 9;24:639. doi: 10.1186/s12891-023-06773-4 (PMC10410998; doi:10.1186/s12891-023-06773-4)
Supplement: Supplementary file 1 — Supplementary Material 1 [file 12891_2023_6773_MOESM1_ESM.docx]

Supplement table 3. Description and comparisons of the groups: No pain, Low PPTs, CWP, and Low PPTs and CWP, respectively

|  | Missing | No pain  Median (IQR) | Low PPTs  Median (IQR) | CWP  Median (IQR) | Low PPTs and CWP  Median (IQR) | p-value |
| --- | --- | --- | --- | --- | --- | --- |
| N |  | 183 | 27 | 30 | 10 |  |
| Age, year | 0/0/0/0 | 54 (11) | 54 (11) | 58 (8) | 49 (16) | 0.049 |
| Sex, female n (%) | 0/0/0/0 | 125 (68) | 16 (59) | 27 (90) | 9 (90) | 0.024 |
| BMI, kg/m^2^ | 0/0/0/0 | 24.9 (5.4) | 27.0 (6.0) | 27.4 (8.0) | 28.0 (8.3) | 0.046 |
| VFA, cm^2^ | 3/0/1/0 | 90 (60) | 125 (70) | 124 (105) | 156 (86) | 0.002 |
| HbA1c, mmol/mol | 6/2/0/0 | 36 (5) | 37 (6) | 38 (4) | 36 (5) | 0.612 |
| Glucose, mmol/L | 1/0/1/0 | 5.4 (0.7) | 5.5 (0.7) | 5.5 (0.7) | 5.1 (0.9) | 0.318 |
| Raised glucose* n (%) | 1/0/1/0 | 63 (35) | 12 (44) | 12 (41) | 3 (30) | 0.684 |
| Triglycerides, mmol/L | 0/0/0/0 | 0.8 (0.5) | 0.8 (0.4) | 1.1 (0.8) | 1.4 (1.6) | 0.108 |
| Raised triglycerides* n (%) | 0/0/0/0 | 22 (12) | 3 (11) | 7 (23) | 5 (50) | 0.005 |
| Cholesterol, mmol/L | 0/0/0/0 | 5.2 (1.5) | 5.1 (1.2) | 5.6 (1.8) | 4.3 (1.2) | 0.123 |
| HDL-Cholesterol, mmol/L | 0/0/0/0 | 1.7 (0.6) | 1.5 (0.6) | 1.6 (0.6) | 1.5 (0.3) | 0.076 |
| Reduced HDL-Cholesterol* n (%) | 0/0/0/0 | 19 (10) | 4 (15) | 3 (10) | 5 (50) | 0.003 |
| LDL-Cholesterol, mmol/L | 0/0/0/0 | 3.4 (1.3) | 3.2 (1.3) | 3.7 (1.8) | 3.1 (1.1) | 0.392 |
| Leptin, ng/ml | 0/0/0/0 | 11.7 (19.0) | 17.8 (26.6) | 21.8 (56.2) | 32.7 (74.3) | 0.001 |
| CRP, mg/L | 1/0/0/0 | 1.1 (1.1) | 1.2 (1.7) | 1.4 (1.6) | 1.6 (2.9) | 0.525 |

Body mass index, BMI; visceral fat area, VFA; haemoglobin A1c, HbA1c; high-density lipoprotein, HDL; low-density lipoprotein, LDL; C-reactive protein, CRP; knee injury and osteoarthritis outcome, KOOS

*According to International Diabetes Federation. [36]
