# Supplementary material for: Associations between chronic widespread pain, pressure pain thresholds, leptin, and metabolic factors in individuals with knee pain
Source: BMC Musculoskelet Disord. 2023 Aug 9;24:639. doi: 10.1186/s12891-023-06773-4 (PMC10410998; doi:10.1186/s12891-023-06773-4)
Supplement: Supplementary file 2 — Supplementary Material 2 [file 12891_2023_6773_MOESM2_ESM.docx]

Supplement table 4. Associations with having low PPTs and reporting CWP analysed with two logistic regression models. The multivariate logistic regression model was adjusted for age and sex.

|  | Univariate | | | Multivariate | |  |  |
| --- | --- | --- | --- | --- | --- | --- | --- |
|  | OR | 95% CI | p-value | OR | 95% CI | p-value | |
| Age, year | 0.932 | 0.874-0.993 | 0.031 |  |  |  | |
| Sex, female | 3.857 | 0.480-31.0 | 0.204 |  |  |  | |
| BMI, kg/m^2^ | 1.077 | 0.963-1.205 | 0.194 | 1.105 | 0.991-1.231 | 0.073 | |
| VFA, cm^2^ | 1.010 | 1.000-1.021 | 0.050 | 1.013 | 1.002-1.024 | 0.025 | |
| Raised triglycerides* | 6.500 | 1.282-23.7 | 0.005 | 12.4 | 2.792-55.0 | 0.001 | |
| Cholesterol, mmol/L | 0.489 | 0.243-0.983 | 0.045 | 0.593 | 0.283-1.242 | 0.166 | |
| Reduced HDL-Cholesterol* | 8.231 | 2.233-30.3 | 0.002 | 8.7 | 2.233-34.2 | 0.002 | |
| Leptin, ng/ml | 1.027 | 1.009-1.044 | 0.002 | 1.032 | 1.011-1.053 | 0.002 | |

Body mass index, BMI; visceral fat area, VFA; hemoglobin A1c, HbA1c; high-density lipoprotein, HDL; low-density lipoprotein, LDL; C-reactive protein, CRP.

*According to International Diabetes Federation [36]
