# Supplementary figures and images for: Associations between chronic widespread pain, pressure pain thresholds, leptin, and metabolic factors in individuals with knee pain
Source: BMC Musculoskelet Disord. 2023 Aug 9;24:639. doi: 10.1186/s12891-023-06773-4 (PMC10410998; doi:10.1186/s12891-023-06773-4)

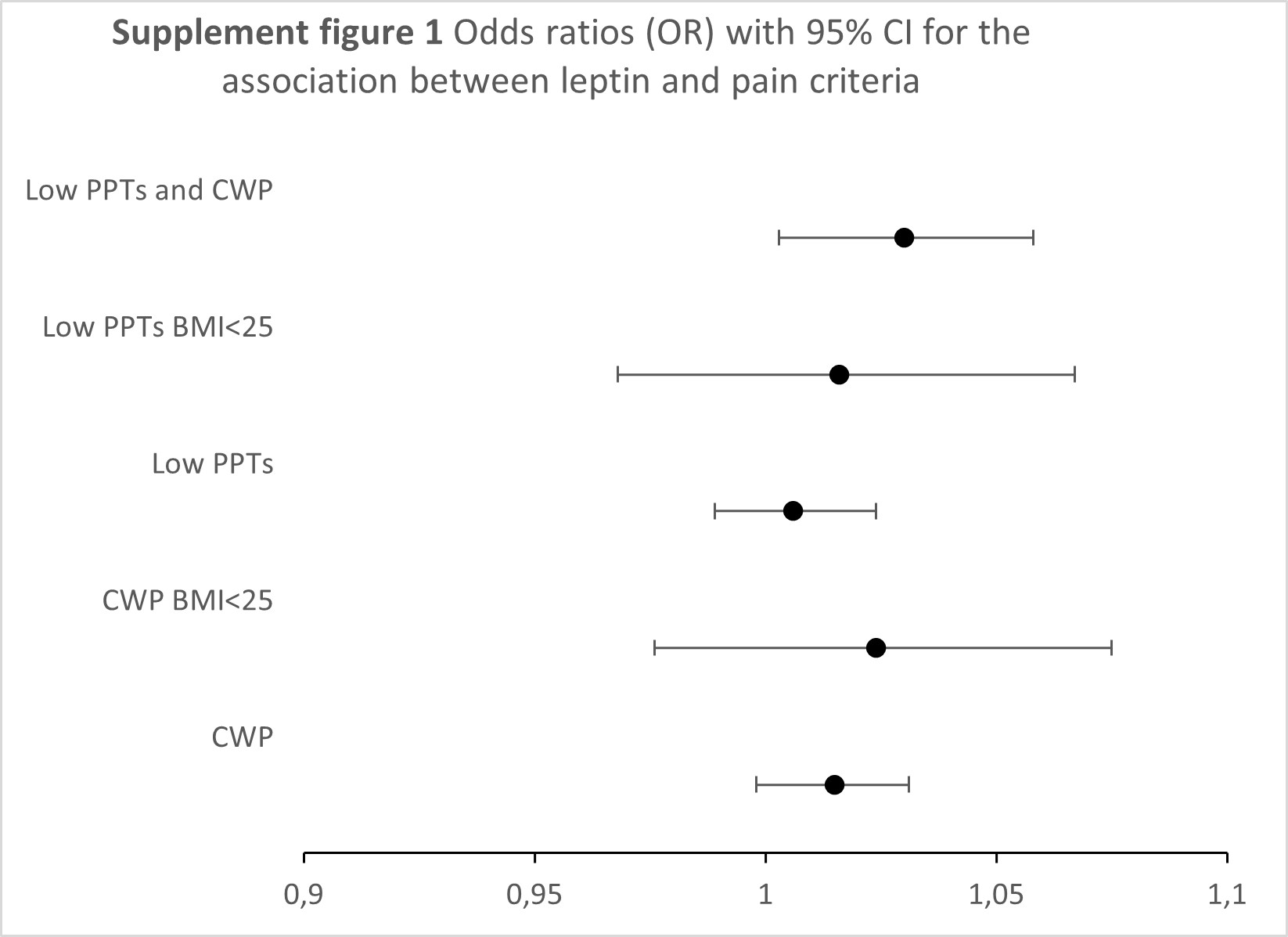

Supplement: Supplementary file 3 — Supplementary Material 3 [file 12891_2023_6773_MOESM3_ESM.jpg]
