# Supplementary material for: Associations between chronic widespread pain, pressure pain thresholds, leptin, and metabolic factors in individuals with knee pain
Source: BMC Musculoskelet Disord. 2023 Aug 9;24:639. doi: 10.1186/s12891-023-06773-4 (PMC10410998; doi:10.1186/s12891-023-06773-4)
Supplement: Supplementary file 4 — Supplementary Material 4 [file 12891_2023_6773_MOESM4_ESM.docx]

|  | Univariate | | | Multivariate* | |  |  |
| --- | --- | --- | --- | --- | --- | --- | --- |
|  | OR | 95% CI | p-value | OR | 95% CI | p-value | |
| Age, year | 0.992 | 0.937-1.050 | 0.779 |  |  |  | |
| sex, female n (%) | 3.833 | 0.476-30.882 | 0.207 | 3.845 | 0.477-30.99 | 0.206 | |
| VFA, cm^2^ | 1.022 | 1.003-1.042 | 0.020 | 1.021 | 1.001-1.040 | 0.035 | |
| Raised triglycerides* | 27.273 | 2.608-285.1 | 0.006 | 23.067 | 2.181-243.9 | 0.009 | |
| Reduced HDL kolesterol* | 6.614 | 1.307-33.47 | 0.022 | 6.482 | 1.207-34.80 | 0.029 | |
| Leptin, ng/mL | 1.040 | 1.006-1.076 | 0.021 | 1.036 | 1.002-1.071 | 0.038 | |

Supplement table 1 Associations to reporting CWP in the subgroup with normal BMI (BMI<25kg/m^2)^. The multivariate logistic regression model was adjusted for age and sex.

Body mass index, BMI; visceral fat area, VFA; haemoglobin A1c, HbA1c; high-density lipoprotein, HDL; low-density lipoprotein, LDL; C-reactive protein, CRP

*According to International diabetes federation (IDF) [36]
