# Supplementary material for: Associations between chronic widespread pain, pressure pain thresholds, leptin, and metabolic factors in individuals with knee pain
Source: BMC Musculoskelet Disord. 2023 Aug 9;24:639. doi: 10.1186/s12891-023-06773-4 (PMC10410998; doi:10.1186/s12891-023-06773-4)
Supplement: Supplementary file 5 — Supplementary Material 5 [file 12891_2023_6773_MOESM5_ESM.docx]

|  | Univariate | | | Multivariate* | |  |  |
| --- | --- | --- | --- | --- | --- | --- | --- |
|  | OR | 95% CI | p-value | OR | 95% CI | p-value | |
| Age, year | 0.955 | 0.905-1.009 | 0.100 |  |  |  | |
| sex, female n (%) | 0.824 | 0.208-3.260 | 0.782 |  |  |  | |
| BMI, kg/m^2^ | 1.250 | 0.957-1.634 | 0.102 | 1.239 | 0.944-1.625 | 0.122 | |
| VFA, cm^2^ | 1.016 | 0.998-1.034 | 0.086 | 1.019 | 0.999-1.039 | 0.057 | |
| Raised triglycerides * | 9.455 | 1.210-73.893 | 0.032 | 11.28 | 1.318-96.50 | 0.027 | |
| Reduced HDL kolesterol* | 3.673 | 0.636-21.218 | 0.146 | 3.019 | 0.493-18.48 | 0.232 | |
| Leptin, ng/mL | 1.028 | 0.997-1.060 | 0.073 | 1.030 | 0.997-1.065 | 0.073 | |

Supplement table 2 Associations to having low PPT in the subgroup with normal BMI (BMI<25kg/m^2^). The multivariate logistic regression model adjusted for age and sex

Body mass index, BMI; visceral fat area, VFA; haemoglobin A1c, HbA1c; high-density lipoprotein, HDL; low-density lipoprotein, LDL; C-reactive protein, CRP

*According to International diabetes federation (IDF) [36]
